# GTSE1: a novel TEAD4-E2F1 target gene involved in cell protrusions formation in triple-negative breast cancer cell models

**DOI:** 10.18632/oncotarget.18691

**Published:** 2017-06-27

**Authors:** Debora Stelitano, Yamila Peche Leticia, Emiliano Dalla, Martin Monte, Silvano Piazza, Claudio Schneider

**Affiliations:** ^1^ Laboratorio Nazionale del Consorzio Interuniversitario per le Biotecnologie (L.N.CIB), Trieste, Italy; ^2^ Laboratorio de Oncología Molecular, Departamento de Química Biológica and IQUIBICEN-UBA/CONICET, Universidad de Buenos Aires, Buenos Aires, Argentina; ^3^ Bioinformatics Core facility, Centre for Integrative Biology, University of Trento (CIBIO), Trento, Italy; ^4^ Dipartimento di Scienze Biomediche e Biologiche (DSMB), Università degli Studi di Udine, Udine, Italy

**Keywords:** GTSE1, TEAD4, E2F1, YAP/TAZ, cell protrusions

## Abstract

GTSE1 over-expression has been reported as a potential marker for metastasis in various types of malignancies, including breast cancer. Despite this, the transcriptional regulation of this protein and the causes of its misregulation in tumors remain largely unknown. The aims of this work were to elucidate how GTSE1 is regulated at the transcriptional level and to clarify the mechanism underlying GTSE1-dependent cell functions in triple-negative breast cancer (TNBC).

Here, we identified GTSE1 as a novel target gene of the TEAD4 transcription factor, highlighting a role for the YAP and TAZ coactivators in the transcriptional regulation of GTSE1.

Moreover, we found that TEAD4 controls the formation of cell protrusions required for cell migration through GTSE1, unveiling a relevant effector role for this protein in the TEAD-dependent cellular functions and confirming TEAD4 role in promoting invasion and metastasis in breast cancer.

Finally, we highlighted a role for the pRb-E2F1 pathway in the control of GTSE1 transcription and observed that treatment with drugs targeting the pRb-E2F1 or YAP/TAZ-TEAD pathways dramatically downregulated the expression levels of GTSE1 and of other genes involved in the formation of metastasis, suggesting their potential use in the treatment of TNBC.

## INTRODUCTION

Breast cancer is one of the most frequently diagnosed forms of cancer and the second leading cause of death in Western women [1]. It is a genetically and clinically heterogeneous disease [2], so no standard therapy is available for all the possible subtypes. About 15% of all invasive breast tumours are triple-negative breast cancers (TNBC) [3]. These cancers seem to be more aggressive than other breast cancer subtypes, leading to a high recurrence probability and poor survival rates [2].

In fact, the lack of hormone receptors and the absence of amplification of the HER2 gene make TNBC insensible to hormonal and anti-HER2 therapies, this leading to a difficult treatment scheme and to the need of identifying novel therapeutic targets for a more target-directed approach.

In a previous study, we reported that GTSE1 (G2 and S phase expressed 1) expression was up-regulated in breast cancer, especially in TNBC [4]. GTSE1 is a cell cycle regulated, microtubule-associated protein whose expression in non-transformed cells is very low during G1, raises during the S phase and peaks during the G2 phase of the cell cycle [5–7]. On the contrary, in transformed cells GTSE1 protein levels are elevated across all the cell cycle phases [4].

In the last years, the number of evidence supporting a strong connection between GTSE1 misregulation and cancer progression has remarkably increased. We and others reported that GTSE1 promotes different aspects of cancer progression including chemoresistance, chromosome instability (CIN) and metastasis [4, 8–13]. In particular, GTSE1 silencing increases the sensitivity of cancer cells to paclitaxel and cisplatin-induced cell death while in highly CIN cancer cell lines it diminishes the defects in chromosome segregation [8–10].

We previously demonstrated that GTSE1 is able to promote focal adhesions disassembly and cancer cells migration, two main features of cells that have acquired metastatic capabilities [4]. Moreover, GTSE1 expression levels associate with invasive potential and tumour grade, with higher protein levels in the most aggressive and invasive breast cancer cell lines. In fact, patients with breast cancer and higher GTSE1 levels show shorter time to distant metastasis and shorter survival time [4].

Interestingly, GTSE1 up-regulation was identified as a potential marker for metastasis not only in breast cancer but also in gastroenteropancreatic neuroendocrine tumor, oral tongue squamous cell carcinoma and hepatocellular carcinoma [11–13].

In spite of the rising interest about GTSE1 and its role in cancer progression, its transcriptional regulation and the causes of its deregulation in cancer remain poorly understood.

Given these reasons, the goal of this work was to unveil the pathways responsible for the control of GTSE1 expression, identifying the transcription factors (TFs) and the coactivators involved and to elucidate the mechanism underlying GTSE1-dependent cell movement in TNBC.

Taking advantage of a multidisciplinary approach, we unveiled a key role for the TEAD4 and E2F1 transcription factors in the transcriptional regulation of GTSE1.

In mammals, the TEAD family of transcription factors includes four highly conserved proteins, named TEAD1-4, that share a common N-terminal TEA DNA binding domain and a C-terminal domain involved in transactivation [14]. However, the TEAD TFs lack a real transcription activation domain and require the interaction with coactivators to promote the transcription of target genes [15]. In fact, coactivators possess an activation domain that allows them to interact with the basal transcription and chromatin remodeling machinery [16]. The interaction between the TEAD TFs and their coactivators is mediated by the TEAD C-terminal domain.

TEAD-interacting coactivators comprise the protein YAP (Yes-associated protein), its homolog TAZ, the family of p160 coactivators and the Vgll proteins [16]. The TEAD family members are the main partners of the YAP and TAZ transcriptional coactivators in the control of epithelial to mesenchymal transition, cancer progression and metastasis [17–19], controlling in particular breast cancer cells migration and invasion [18, 19]. YAP and TAZ (also known as WWTR1, WW domain-containing transcription regulator protein1) are the downstream effectors of the Hippo signaling transduction pathway, a tumor suppressor pathway frequently deregulated in cancers. Here, we demonstrated that the YAP/TAZ-TEAD4 axis plays a pivotal role in the transcriptional regulation of GTSE1, showing that the effect of TEAD on cell migration and invasion is at least partially GTSE1-dependent. Moreover, we showed that TEAD regulates the formation of cell protrusions necessary for cancer cells migration through GTSE1 providing, for the first time, a mechanistic explanation of how it affects cell migration.

However, the YAP/TAZ-TEAD axis is not the only pathway involved in the transcriptional regulation of GTSE1. In fact, in the present work we also highlighted a role for the pRb-E2F1 pathway in the control of GTSE1 transcription.

E2F1 is the founding member of the E2F family of transcription factors that comprises eight proteins (E2F1-E2F8) playing a key role in cellular processes such as cell cycle progression, DNA repair, apoptosis, chromosome stability and development [20–24]. In particular, E2F1 fulfills one of its crucial regulatory roles by modulating the progression of cell cycle from the G1 to the S phase through the control of the transcription of its target genes and, for this reason, its expression and activity must be tightly regulated [21, 25–27]. The main negative regulator of E2F1 activity is the retinoblastoma protein (pRb) [28] and the deregulation of the CDKs/pRb/E2F1 network may result in tumorigenesis.

Here, we demonstrated that E2F1 is able to bind the GTSE1 promoter and to regulate its expression. In fact, the silencing of this transcription factor or the pharmacological inhibition of the pRb-E2F1 pathway dramatically decreases GTSE1 transcription, strongly indicating the involvement of this pathway in the regulation of its expression.

## RESULTS

### Regulation of GTSE1 expression by the YAP/TAZ-TEAD axis

**Figure 1:**
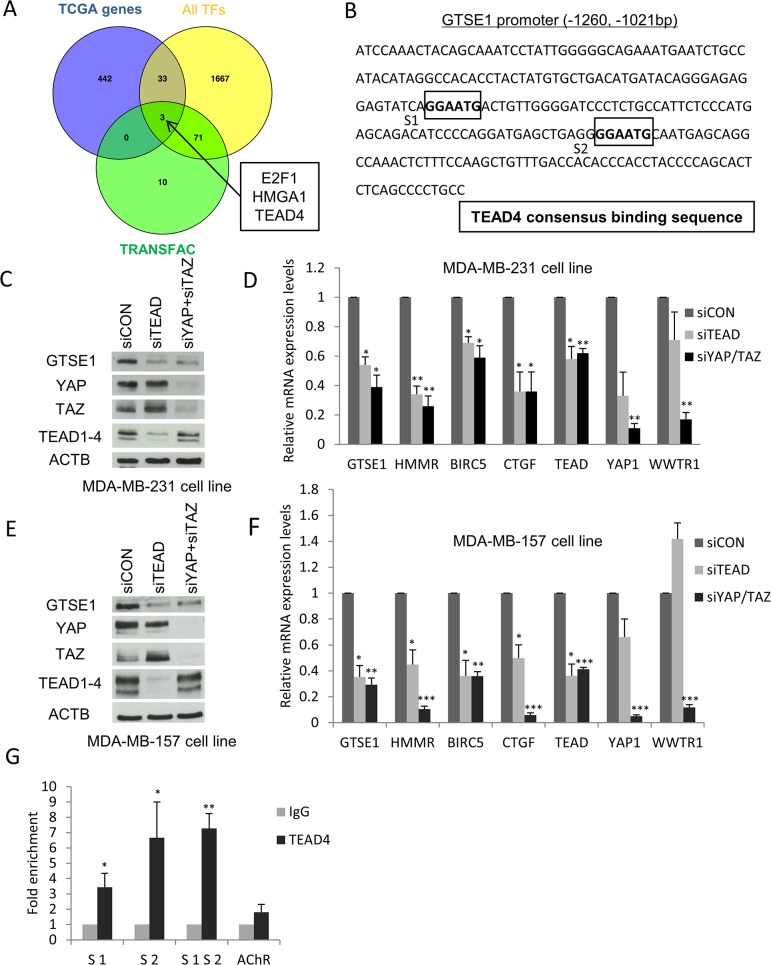
Identification of TEAD, E2F1 and HMGA1 transcription factors as novel regulators of GTSE1 expression **(A)** Comparison of the TCGA-derived list of GTSE1 co-expressed genes (n=478) with TRANSFAC/Match results (n=84) and with the curated list of human known TFs (n=1,774). Among the 36 TFs co-expressed with GTSE1, TRANSFAC/Match only predicted the presence of TFBS for E2F1, HMGA1 and TEAD4 in the GTSE1 promoter. **(B)** Mapping of the TEAD4 binding sites in the GTSE1 promoter region. **(C-F)** The TEAD1/3/4 or YAP/TAZ knockdown markedly reduced GTSE1 expression levels. GTSE1 protein and mRNA levels after TEAD1/3/4 or YAP/TAZ silencing by siRNA in MDA-MB-231 (C and D) and MDA-MB-157 cell lines (E and F). Data are presented as mean ± SEM of three independent experiments. For the statistical analysis, Student two-tailed t-test was applied. *p-value<0.05; **p-value<0.01; *** p-value<0.001. **(G)** TEAD4 physically binds the GTSE1 promoter region. Anti-IgG (control) or anti-TEAD4 antibodies were used in ChIP assays on the MDA-MB-231 cell line. The human muscarinic receptor gene was used as negative control (AChR). Data are presented as mean ± SEM of three independent replicates. For statistical analysis, Student's t-test was applied. *p-value<0.05; **p-value<0.01.

The first objective of our work was to elucidate how the GTSE1 protein was regulated at the transcriptional level and the transcription factors involved. Hence, taking advantage of published TCGA breast cancer gene expression data, we performed a bioinformatics analysis in order to identify the transcription factors co-expressed with GTSE1, expecting that the list of 36 TFs that we obtained should comprise, among others, the modulators of GTSE1 expression. In order to obtain the best candidates to the role of regulators of GTSE1 transcription, we overlapped these results with the output generated by the TRANSFAC/Match tool [29], listing the TFs exhibiting at least one binding site (TFBS) in the genomic region corresponding to the GTSE1 promoter. Interestingly, the outcome showed a very short TFs list including TEAD, E2F1 and the chromatin modifier HMGA1 (Figure 1A).

Initially, we focused our attention on the regulation of GTSE1 expression by the TEAD family of TFs. We mapped the TEAD consensus binding sequence (“GGAATG”) on the GTSE1 promoter, finding that it harbored two putative binding sites located 1162 and 1097 bp upstream from the transcription start site (TSS) (Figure 1B). This preliminary result strengthened the possibility for the TEAD family to play a role in the regulation of GTSE1 expression. To test this hypothesis, we evaluated the effect of TEAD1/3/4 knockdown on GTSE1 expression levels using a siRNA to silence TEAD1/3/4 in the MDA-MB-231 cell line and assessing GTSE1 protein levels after 72 hours. Under these conditions, GTSE1 expression levels dramatically decreased confirming our previous assumption (Figure 1C).

Since the lack of a real activation domain in TEAD family members requires the interaction with the YAP and TAZ coactivators to promote the transcription of target genes, we assessed the effect of YAP/TAZ silencing on GTSE1 protein levels. As shown in Figure 1C, GTSE1 expression significantly dropped after the double silencing compared to the control.

To clarify if the YAP/TAZ-TEAD axis exerted its regulation on GTSE1 at the transcriptional level, we silenced TEAD1/3/4 and YAP/TAZ using siRNAs in the MDA-MB-231 cell line and evaluated GTSE1 mRNA levels after 72 hours. As shown in picture Figure 1D, GTSE1 mRNA levels dramatically diminished in cells silenced for TEAD1/3/4 and YAP/TAZ. As positive controls, we measured the mRNA levels of the known YAP/TAZ-TEAD target genes BIRC5, HMMR and CTGF. The knockdown of YAP/TAZ and TEAD significantly decreased the mRNA levels of all these target genes.

To evaluate if other TNBC cell lines shared the same regulation of GTSE1 expression mediated by the YAP/TAZ-TEAD axis, we carried out TEAD1/3/4 and YAP/TAZ silencing in the MDA-MB-157 cell line and measured the levels of the GTSE1 protein (Figure 1E) and mRNA (Figure 1F). Under this experimental setting, GTSE1 expression levels significantly decreased in silenced cells compared to the control ones, confirming the results obtained in the MDA-MB-231 cell line. The obtained results suggest that the YAP and TAZ transcriptional coactivators regulate GTSE1 expression at the transcriptional level through their interaction with the TEAD family of transcription factors.

### GTSE1 is a novel target gene of TEAD4

Among the different members of the TEAD family, TEAD4 has been previously shown to be up-regulated in breast cancer cell lines (and in particular in TNBC) and to be able to control the expression of genes involved in breast cancer cell migration and invasion like HMMR [19, 30]. After identifying two putative TEAD4 binding sites in the GTSE1 promoter (Figure 1B), we performed a ChIP assay using an antibody against TEAD4 to assess if this transcription factor physically interacted with that genomic region and to identify its real binding sites. As shown in Figure 1G, TEAD4 occupies both the S1 and S2 binding sites on the GTSE1 promoter. These results demonstrate that GTSE1 is a novel *bona fide* target gene of TEAD4 and suggest that this TF could promote GTSE1 transcription through the direct binding to its promoter region.

### GTSE1 expression is regulated by the Mevalonate Pathway

The mevalonate pathway supports the YAP/TAZ-dependent transcriptional program by promoting their nuclear accumulation and activity [19], [31]. On the contrary, treatment with cerivastatin, a molecule able to block the mevalonate pathway and the cholesterol biosynthesis [32], leads to YAP/TAZ cytoplasmic retention, stopping the transcription of their target genes [31].

Based on this evidence, we tested the effect of the cerivastatin-induced inhibition of the mevalonate pathway on GTSE1 expression.

**Figure 2:**
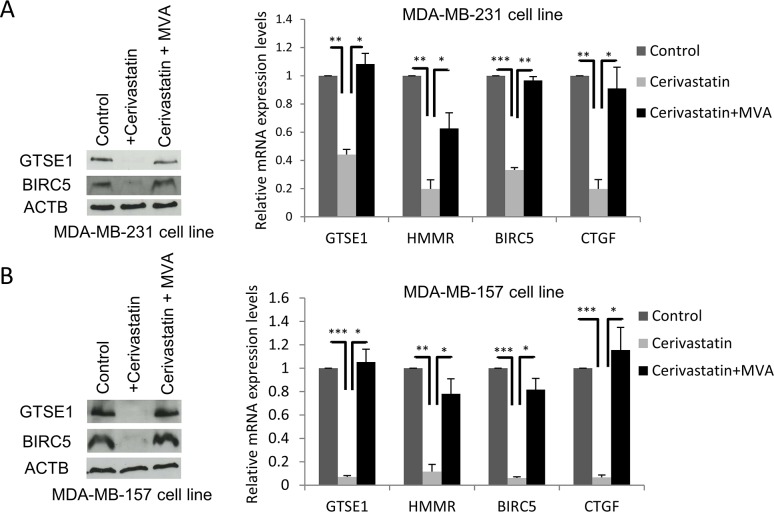
The Mevalonate pathway regulates GTSE1 expression **(A and B)** The inhibitor of the Mevalonate pathway cerivastatin significantly decreased GTSE1 expression levels, whereas the addition of mevalonate (MVA) completely abolished its inhibitory effect. (A) GTSE1 protein and mRNA levels after treatment with cerivastatin 1μM alone or in combination with MVA 0.5mmol for 24 hours in MDA-MB-231 cell line. (B) GTSE1 protein and mRNA levels in MDA-MB-157 cell line treated as in *A* for 72 hours. Data are presented as mean ± SEM of three independent replicates. Student two-tailed t-test was applied for the statistical analysis. *p-value<0.05; **p-value<0.01; ***p-value<0.001.

MDA-MB-231 and MDA-MB-157 cell lines were treated with cerivastatin 1μM and GTSE1 expression levels were assessed. As shown in Figure 2, GTSE1 protein and mRNA levels dramatically decrease in cerivastatin treated cells with respect to control cells (Figure 2A and 2B). The addition of mevalonate to cerivastatin treated cells, promoting YAP/TAZ nuclear localization and activity, is able to completely rescue the effect of cerivastatin (Figure 2A and 2B).

These results indicate that the mevalonate pathway regulates the expression of GTSE1, as shown for other YAP/TAZ targets, further suggesting the involvement of these transcriptional coactivators in the control of GTSE1 transcription.

### TEAD4 regulates breast cancer cells migration through GTSE1

YAP, TAZ and TEAD4 are well-known regulators of breast cancer cell migration and invasion. In fact, the ability of TNBC cell lines to migrate and to invade decreases after TEAD, YAP and TAZ silencing [19, 33]. As mentioned, GTSE1 activity is another feature required for breast cancer cells migration [4]. Consequently, we investigated if the effect of TEAD on cell migration and invasion was mediated by GTSE1.

**Figure 3:**
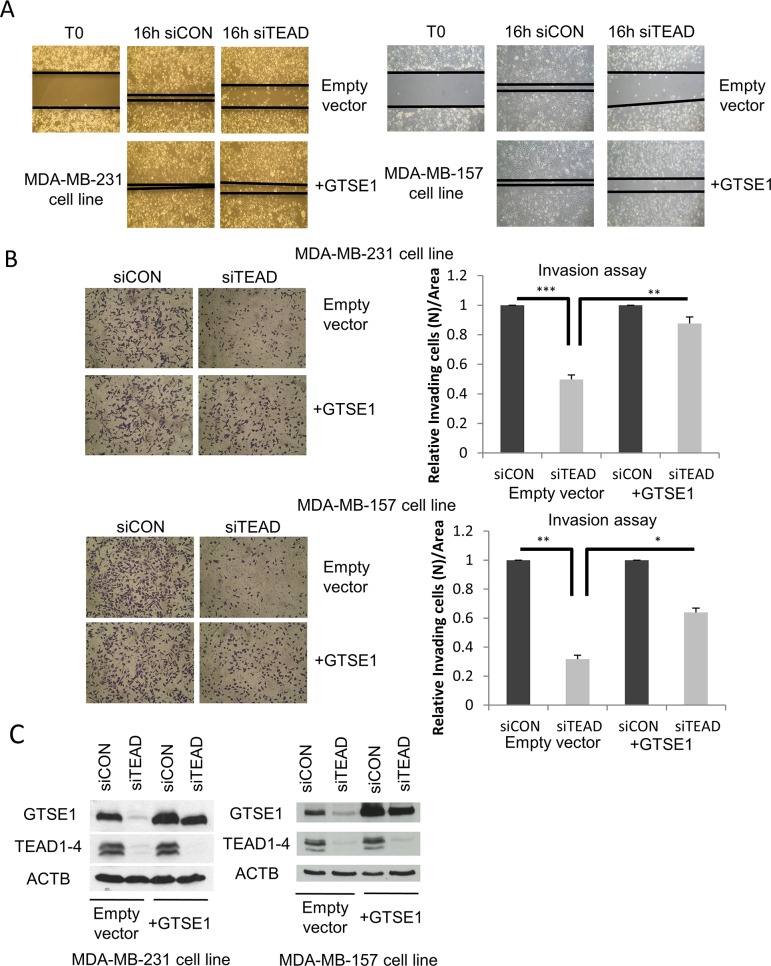
TEAD regulates breast cancer cell migration and invasion through GTSE1 **(A)** Representative images of the wound-healing motility assays showing the capability of GTSE1 to rescue the reduced cell migration followed to TEAD1/3/4 silencing. The scratch assay was carried out in MDA-MB-231 (left) and MDA-MB-157 cell lines (right) containing a stably integrated GTSE1 over-expressing construct (pBABE-GTSE1) or an empty vector (pBABE). **(B)** Representative images of transwell invasion assays showing the ability of GTSE1 to rescue the reduced cell invasion followed to TEAD1/3/4 knockdown. The transwell invasion assays were performed in the same cell lines used for the wound-healing assay. Histograms show the mean number of cells/area that invaded through the transwell inserts after 18 h. Error bars represent the standard error of the mean from three independent experiments. Student two-tailed t-test was applied for statistical analysis. *p-value<0.05; **p-value<0.01; ***p-value<0.001. **(C)** Western blot of the MDA-MB-231 and MDA-MB-157 cell lines containing a stably integrated construct over-expressing GTSE1 (pBABE-GTSE1) or empty vector (pBABE).

As shown in Figure 3, over-expression of GTSE1 is able to rescue the reduced ability of TEAD-silenced TNBC cell lines to migrate in wound healing and transwell migration assay (Figure 3A and Supplementary Figure 1), and to invade (Figure 3B) as measured through transwell invasion assay, with no statistically significant difference in the total number of cells in the considered time interval (Supplementary Figure 2).

These results indicate that the effect of TEAD on cell migration and invasion is GTSE1-dependent, unveiling a relevant effector role for GTSE1 in TEAD-dependent cellular functions.

We next investigated the mechanism by which TEAD controls cell migration through GTSE1.

The establishment of a front-back cell polarity is required for cell migration of mesenchymal-like cells [34]. The front is characterized by F-actin rich filaments, called cell protrusions, that allow the cell to extend forward to adhere to the substrate, while the rear is retractile and generates the force necessary to push up the cell body in the direction of the movement [35].

**Figure 4:**
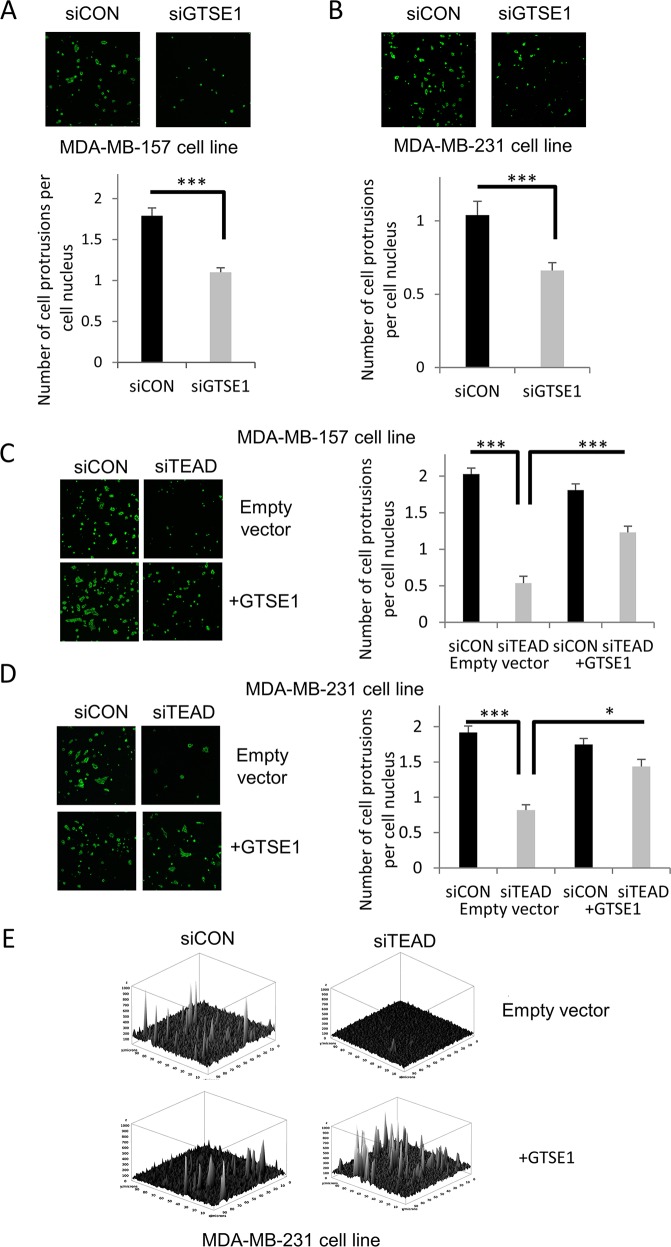
TEAD4 modulates the formation of cell protrusions through GTSE1 **(A and B)** GTSE1 knockdown significantly reduced the formation of cell protrusions. Representative images of cell protrusions stained for F-actin and histograms representing the number of cell protrusions per cell nucleus after control treatment or GTSE1 silencing in MDA-MB-157 (A) and MDA-MB-231 cell lines (B). **(C and D)** TEAD silencing dramatically decreased the cell protrusions formation, whereas GTSE1 overexpression partially abolished this effect. The cell protrusions assays were performed in MDA-MB-157 and MDA-MB-231 cell lines containing an empty vector (pBABE) or a stably integrated GTSE1 over-expressing construct (pBABE-GTSE1) and cell protrusions were stained as in *A* and *B*. Histograms show the number of cell protrusions per cell nucleus after control treatment or TEAD silencing. All data of this figure are presented as mean ± SEM of three independent experiments. For statistical analysis, Student two-tailed t-test was applied. *p-value<0.05; ***p-value<0.001. **(E)** Three-dimensional representation of the cell protrusions (MDA-MB-231) showing the differences between conditions also in term of cell surface extensions.

Since cell protrusions represent a common feature of moving cells in tumors, we wondered if GTSE1 controlled breast cancer cells migration through the regulation of cell protrusions formation. As shown in Figure 4A and 4B, the knockdown of GTSE1 by siRNA reduces the number of cell protrusions per cell.

Afterwards, we evaluated if TEAD regulated the formation of GTSE1-dependent cell protrusions. As shown in Figure 4C, 4D and 4E, TEAD silencing impacts negatively on the number of cell protrusions per cell, while GTSE1 over-expression is able to rescue this effect.

The obtained results suggest that TEAD controls the formation of cell protrusions through GTSE1, providing, for the first time, a mechanical explanation of how it regulates breast cancer cell migration.

### The E2F1 transcription factor controls the expression of the GTSE1 protein

**Figure 5:**
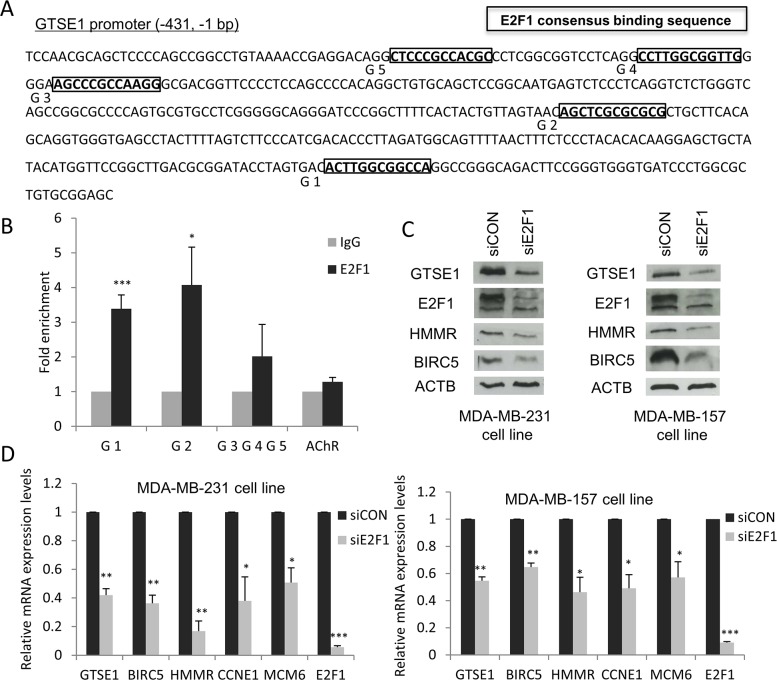
E2F1 modulates GTSE1 expression **(A)** Mapping of the E2F1 binding sites in the GTSE1 promoter region. **(B)** Results of the ChIP assays showing that E2F1 physically binds the G1 and G2 sites in GTSE1 promoter. Anti-IgG (control) or anti-E2F1 antibodies were used in ChIP assays on the MDA-MB-231 cell line. AChR was used as negative control. Data are presented as mean ± SEM of at least three independent replicates. For statistical analysis, Student's t-test was applied. *p-value<0.05; ***p-value<0.001. **(C and D)** GTSE1 expression levels significantly decreased after E2F1 silencing. GTSE1 protein (C) and mRNA levels (D) after knockdown of E2F1 in MDA-MB-231 and MDA-MB-157 cell lines. Data are presented as mean ± SEM of three independent experiments. For statistical analysis, Student two-tailed t-test was applied. *p-value<0.05; **p-value<0.01; ***p-value<0.001.

As mentioned above, TEAD4 is not the only possible transcription factor regulating GTSE1 expression. In fact, based on the results generated by the TRANSFAC/Match tool, we could identify five instances of the E2F1 consensus binding sequence (“TTTSSCGS”, where S = C/G) in the GTSE1 promoter region, respectively located 58 (G1), 201 (G2), 344 (G3), 360 (G4) and 389 (G5) nucleotides upstream from GTSE1 transcription start site (Figure 5A).

By performing a ChIP analysis using antibodies against E2F1, we found that this TF directly binds the G1 and G2 sites in the GTSE1 promoter region, suggesting its involvement as an additional regulator of GTSE1 transcription (Figure 5B). Therefore, we tested the effect of E2F1 knockdown on GTSE1 expression. As shown in Figure 5C and 5D, after E2F1 depletion both the protein and the mRNA levels of GTSE1, similarly to other known E2F1 target genes (i.e. BIRC5 and HMMR), notably decreased. The obtained results, further confirming the involvement of this TF in the control of GTSE1 expression, identify GTSE1 as a novel E2F1 target gene.

It has been reported that 67% of promoters of YAP/TAZ target genes involved in cell proliferation harbors binding sites for E2F1 [36]. Previous studies, in fact, demonstrated that YAP, TEAD and E2F1 cooperate synergistically for the implementation of a transcriptional program required to bypass the cell cycle exit and to promote cell proliferation both in fruit fly and human [37, 38].

Here, we showed that TEAD and E2F1 regulate the expression of genes involved not only in cell proliferation, such as BIRC5, but also of genes (e.g. GTSE1 and HMMR) involved in other aspects of cancer progression such as migration, invasion and metastasis, further highlighting the importance of E2F1 and TEAD cooperation in cancer.

**Figure 6:**
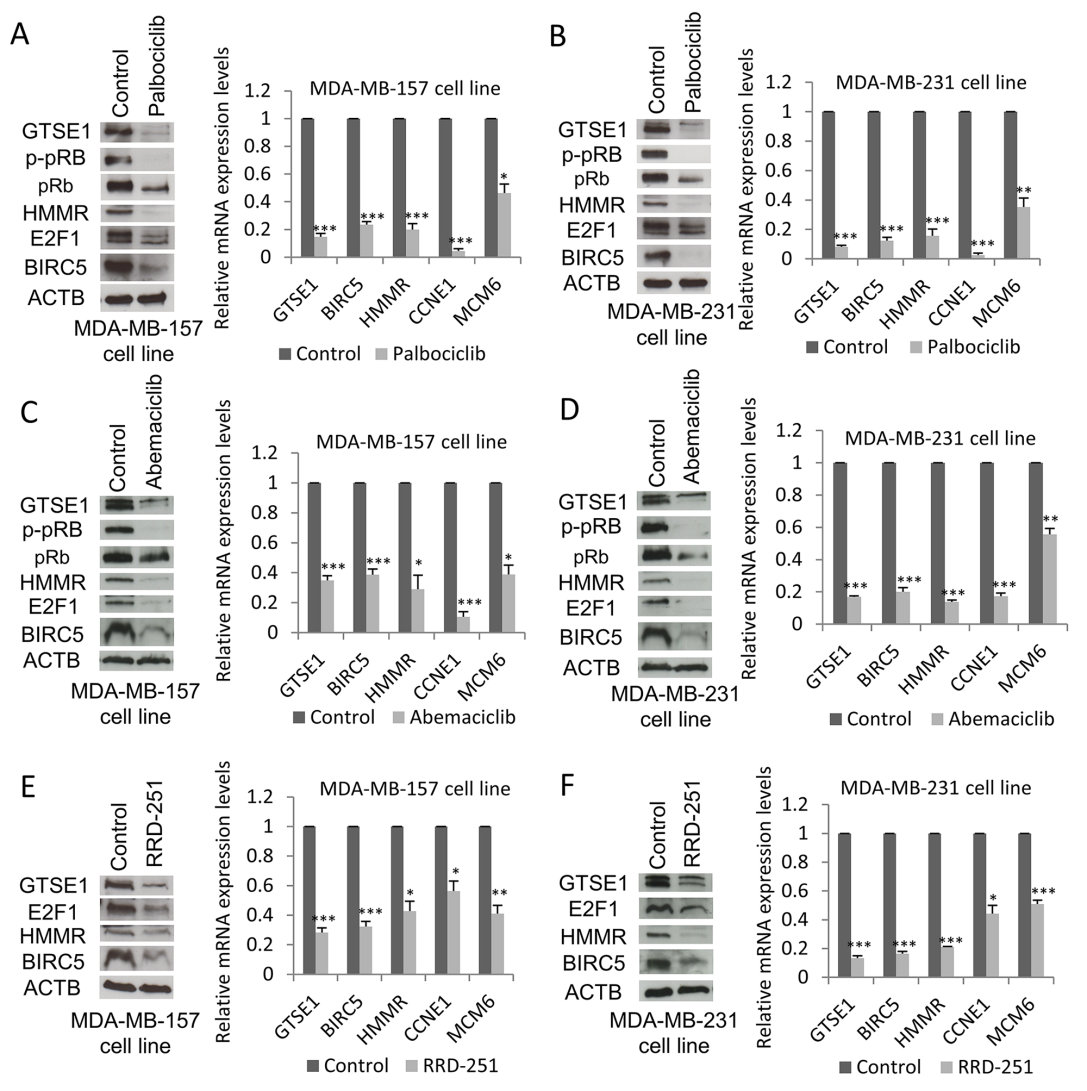
GTSE1 expression levels decrease after treatment with pRb-E2F1 pathway inhibitors GTSE1 protein and mRNA levels in MDA-MB-157 and MDA-MB-231 cell lines treated respectively with: palbociclib 1μM **(A)** and 0.5μM for 24h **(B)**; abemaciclib 0.5μM for 24h **(C and D)**; RRD-251 100μM for 24 hours **(E and F)**. Data are presented as mean ± SEM of three independent experiments. For statistical analysis, Student two-tailed t-test was applied. *p-value<0.05; **p-value<0.01; ***p-value<0.001.

In order to corroborate the GTSE1 regulation by E2F1, we tested the effect of well-known inhibitors of the pRb-E2F1 pathway on GTSE1 expression. Palbociclib (PD0332991) and abemaciclib (LY2835219) are selective inhibitors of CDK4 and CDK6 that cause pRb hypophoshorylation and block of E2F1 activity [39–41]. As shown in Figure 6A, 6B, 6C and 6D, after treatment with these drugs GTSE1 expression levels, as well as other E2F1 target genes (e.g HMMR), dramatically decreased at both protein and mRNA levels. Under these conditions and in both cell lines, total pRb and E2F1 levels concomitantly decrease suggesting dramatic alterations in the pRb-E2F1 pathway.

Moreover, treatment with RRD-251, an inhibitor of pRb-Raf-1 interaction reported to downregulate E2F1 protein levels [42], negatively impacts GTSE1 expression (Figure 6E and 6F).

Taken together, these data further confirm the involvement of the pRb-E2F1 pathway in the transcriptional regulation of GTSE1.

### GTSE1, E2F1 and TEAD4 expression levels in breast cancer subtypes

**Figure 7:**
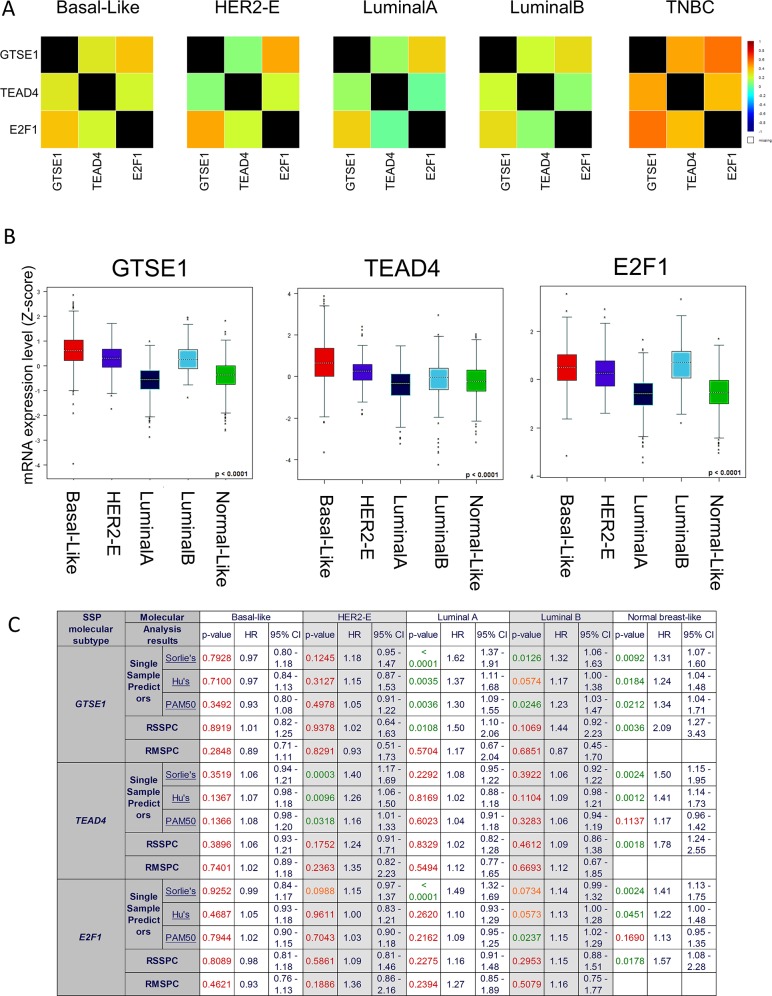
GTSE1, E2F1, TEAD4 Expression levels in Breast Cancer Subtypes **(A)** Correlation heatmaps of the gene expression levels of GTSE1, E2F1, TEAD4 in the different breast cancer subtypes (PAM50 classification). **(B)** Boxplot representation of the gene expression levels of GTSE1, E2F1, TEAD4 in the different breast cancer subtypes (PAM50 classification). **(C)** Univariate cox proportional hazards analyses for GTSE1, E2F1, TEAD4 in the different breast cancer subtypes. Several molecular classifications are presented: Single Sample Predictor (SPP), Robust SSP Classification Robust Molecular (RSSPC) and Robust Molecular Subtype Predictors Classification (RMSPC). P-values were colored accordingly to their statistical significance (green p < 0.05; orange 0.05 < p < 0.1; red p > 0.1).

We previously investigated the relationship between breast cancer clinical variables and GTSE1 expression [4] noticing its association with the most invasive and aggressive cancers (Grade 3). Here we improved the former analysis using GenExMiner [43, 44], one of the most comprehensive collections of breast cancer data sets collectively consisting of more than 5800 patients. In fact, due to the high number of samples, we could perform specific analyses for each subtype. Using the “Gene correlation targeted module” function, the strongest correlation between the genes, based on the expression data, was observed in the TNBC subtype (GTSE1 vs TEAD4 or E2F1, cor=0.45 p<0.0001 and cor=0.55 p<0.0001) (See Figure 7A and Supplementary Figure 3). Similar results were obtained using the “Targeted expression module” function, included in GenExMiner: in this case, the three genes were all more expressed in Basal-like, HER2 enriched and Luminal B subtypes (see Figure 7B and Supplementary Figure 4). While survival analyses (Cox proportional hazards models) for the whole dataset were significant for all the three genes (see Supplementary Figure 5), we observed a complex pattern in the specific subtypes (See Figure 7C and Supplementary Figure 6): only in Normal-like and Luminal A subsets, in fact, we could obtain partially statistically significant results. This is nevertheless expected, since in Basal-like, HER2 enriched and Luminal B subtypes the expression of the three genes is at high level in almost all samples, making it difficult to stratify the patients.

## DISCUSSION

In the last years, the involvement of the TEAD family of transcription factors together with the YAP and TAZ coactivators has strongly emerged in the development of different types of tumors, including breast cancer. In fact, both TEAD4 and TAZ over-expression in breast cancer correlates with poor prognosis [30, 45]. Moreover, the YAP and TAZ coactivators interact with the TEAD TFs to promote epithelial to mesenchymal transition, migration and invasion, that are all critical events in cancer progression and metastasis formation [18, 19].

Despite the great interest, less is known about the YAP/TAZ downstream effectors and how they exert their functions.

In this study, using a multidisciplinary approach we identified GTSE1 as a novel YAP/TAZ-TEAD4 regulated protein. The YAP/TAZ coactivators and the TEAD4 transcription factor exert their regulation at the transcriptional level promoting GTSE1 transcription. In fact, both the TEAD and the YAP/TAZ knockdown lead to a decrease of GTSE1 mRNA level. Moreover, ChIP experiments confirmed the presence of TEAD4 on the GTSE1 promoter, suggesting that it could directly control GTSE1 expression.

We had previously reported that GTSE1 is required for TNBC cell migration and another study had also demonstrated that YAP, TAZ and TEAD down-regulation impacts negatively on the ability of breast cancer cells to migrate [19]. For these reasons, we verified if TEAD controlled breast cancer cells migration through GTSE1.

Here, we found that GTSE1 expression is able to rescue the reduced migration following TEAD silencing in TNBC cell lines, suggesting that the effect of TEAD on cell motility is, at least partially, GTSE1-dependent.

A common feature required for cell migration is the formation of cell protrusions at the leading edge. Here, we highlighted a role for TEAD and GTSE1 in the control of cell protrusions formation. In fact, the number of cell protrusions/cell markedly decreases after TEAD depletion, with GTSE1 expression rescuing, at least partially, this effect. The achieved results suggest that TEAD regulates cell protrusion formation via GTSE1 providing, for the first time, a mechanical explanation on how it regulates cell migration.

TEAD4, however, is not the only transcriptional regulator of GTSE1 expression; in fact, we found that the GTSE1 promoter region contains also E2F1 and HMGA1 binding sites.

Similarly to TEAD4, E2F1 is required for the transcriptional regulation of GTSE1. Its depletion, in fact, causes the lowering of both GTSE1 mRNA and protein levels. As demonstrated by ChIP assay, E2F1 directly binds the GTSE1 promoter region, further supporting its role in the control of GTSE1 transcription and making GTSE1 a novel E2F1 target gene.

The Rb protein is the main regulator of E2F1 activity. Its signature includes 159 genes that are up-regulated after RB1 deletion or repressed by RB1 activation [46] and most of them are also regulated by E2F. As reported by Ertel et al., the signature associated with RB1 loss presents the highest expression values in ER-negative tumors, reflecting a deep deregulation of RB1 in this type of cancers. It has been shown that GTSE1 is part of the RB1 loss signature [46], further confirming the involvement of the pRb-E2F1 regulated pathway in the control of GTSE1 expression.

Both the pRb-E2F1 and the YAP/TAZ-TEAD4 pathways are deregulated in TNBC and, since GTSE1 expression levels are higher in the most aggressive and invasive breast cancer subtypes, this lead us to speculate that these two pathways could cooperate to promote GTSE1 up-regulation, in combination with other genes.

Similarly to GTSE1, HMMR is regulated by both TEAD4 and E2F1 and has a critical role in breast cancer cell migration [19]. These observations suggest that these two transcription factors may cooperate in orchestrating a transcriptional program of genes involved in breast cancer cell migration, invasion and metastasis. An independent regulation of GTSE1 and HMMR expression by E2F1 and TEAD4 is still possible, although less probable; therefore, further studies are required to verify if they act synergistically or not.

Previous studies suggested that breast cancer cells migration is reduced after treatment with palbociclib in a COX2 mediated manner [47]. However, COX2 up-regulation alone is not able to completely rescue the reduced migration that follows the treatment, suggesting the involvement of other pathways. Here, we report that the palbociclib treatment leads to a reduction of the expression of both GTSE1 and HMMR, suggesting that they may contribute to the reduced breast cancer cell migration.

Moreover, we and other previously highlighted a role for GTSE1 in the promotion of chemoresistance [8, 9]. In fact, GTSE1 depletion rises the sensitivity of cancer cells to paclitaxel and cisplatin treatment [8, 9]. For these reasons, GTSE1 targeting will be further explored in the future as a way to interfere simultaneously with different aspects of cancer progression. This is even more relevant to be considered since GTSE1 is also expressed at high levels in subtypes other than TNBC, such as HER2+ and luminal B breast cancers, as we have shown, and in other tumor types [9, 11–13]. Interestingly, the third regulator of GTSE1 expression that emerged from our bioinformatics analysis is the chromatin modifier HMGA1 (high mobility group AT-Hook protein 1). The HMGA1 protein regulates the transcription of target genes through the architectural remodeling of chromatin, allowing the formation of multi-protein complexes on promoter and enhancer regions [48]. It was reported that GTSE1 is part of the HMGA1 loss signature and that this TF regulates GTSE1 expression at least at the transcriptional level [49]. This suggests an attractive hypothesis in which HMGA1 could mediate the chromatin modification required for GTSE1 transcription. In the future, additional studies will be required to investigate HMGA1 role in GTSE1 expression regulation.

In conclusion, our work allowed us to delve deeper into the understanding of GTSE1 transcriptional regulation: we identified the transcription factors and coactivators involved and we established a role for both the YAP/TAZ-TEAD4 and the pRb-E2F1 pathways in the control of GTSE1 expression. GTSE1 is not the only gene involved in cell migration to be regulated by these two pathways, suggesting that they are profoundly interconnected in regulating processes required for cell proliferation but also for other aspects of tumor progression such as invasion and metastasis. Further work is needed to validate our hypothesis that these two pathways cooperate synergistically in the promotion of a transcriptional program required for metastasis. When this will be confirmed, the combinatorial use of drugs targeting both the YAP/TAZ-TEAD4 and the pRb-E2F1 pathways will be tested for the treatment of TNBC.

## MATERIALS AND METHODS

### Cell culture and chemicals

MDA-MB-231 and MDA-MB-157 cell lines were obtained from ATCC. All cell lines were grown in DMEM with 4.5 g/L glucose (Lonza) and L-glutamine, supplemented with 10% fetal bovine serum (Euroclone), 100 U/ml penicillin and 100 U/ml streptomycin (Lonza) at 37°C in a humidified atmosphere of 5% CO_2_.

Cerivastatin (SML0005, Sigma Aldrich) and DL-mevalonic acid 5-phosphate (79849, Sigma Aldrich,) were prepared in dimethyl sulfoxide at a 10mmol/L concentration and 0.5mol/L, respectively.

Palbociclib (PD0332991, PZ 0199, Sigma Aldrich) and abemaciclib (LY2835219, HY-16297, MedChem express) were dissolved in H_2_O at a 1mmol/L concentration. RRD-251 hydrochloride (R7532, Sigma Aldrich) was resuspended in dimethyl sulfoxide at a 50mmol/L concentration.

For cerivastatin treatment, cells were incubated with DMSO or 1μM cerivastatin alone or with 0.5mmol mevalonic acid (MVA) for 24 hours (MDA-MB-231) or 72 hours (MDA-MB-157).

For palbociclib treatment, cells were treated with 0.5μM (MDA-MB-231) or 1μM (MDA-MB-157) PD0332991 for 24 hours. Treatment with abemaciclib was performed incubating cells with 0.5μM LY2835219 for 24 hours. For RRD-251 treatment cells were incubated with DMSO or 100μM RRD-251 for 24 hours.

### RNA interference

siRNAs were purchased from Eurofins Genomics (sequences shown in Supplementary Table 1).

The AllStars negative control siRNA (Qiagen, 1027281) was used as negative control.

Lipofectamine RNAi-MAX (Invitrogen) was used for siRNAs transfections in antibiotic-free medium according to the manufacturer's instructions. Cells were transfected in reverse using 20nM siRNA. The TEAD1/3/4 and YAP/TAZ silencing was performed by transfecting the siRNAs in MDA-MB-231 and MDA-MB-157 cell lines for 72 hours.

GTSE1 and E2F1 silencing was carried out transfecting cells with siRNAs for 48 hours.

### Western blot analysis and antibodies

After silencing or treatment with drugs, western blot analysis was performed according to the standard procedures.

Antibodies and dilutions used are listed in Supplementary Table 2.

Rabbit antibody against GTSE1 was previously described [4]. Bound primary antibodies were visualized using Pierce ECL Plus (Thermo Scientific) after addition of secondary antibodies.

### RT-qPCR

Total RNA extraction was performed using QIAzol Lysis Reagent (Qiagen) and the Nanodrop spectrophotometer was used to quantify the nucleic acid extracted and to assess its purity. The integrity of total RNA extracted was verified by agarose gel electrophoresis.

500 ng of extracted total nucleic acid were reverse-transcribed into cDNA using a QuantiTect Reverse Transcription Kit (Qiagen), according to the manufacturer's instructions. Briefly, genomic DNA contamination was removed through the incubation of purified RNA with gDNA Wipeout buffer at 42°C for 2 minutes, subsequently RNA was reverse-transcribed at 42°C for 30 minutes using the Quantiscript Reverse Transcriptase, the Quantiscript RT buffer and the RT Primer mix supplied by the kit.

The enzyme was then inactivated by incubation at 95°C for 3 minutes.

The obtained cDNAs were diluted 1:20 and 5μl of each sample in triplicate were loaded in the MicroAmp® Fast 96-Well Reaction Plate (Applied Biosystems) to perform the real-time qPCR with the SYBR Green PCR Master Mix (Applied Biosystems). The RT-qPCR was carried out using a StepOnePlus real time PCR machine (Applied Biosystems).

Oligonucleotides were used at a final concentration of 5μM (sequences are shown in Supplementary Table 3). Each experiment was performed at least three times and expression levels were normalized to the ACTB and GAPDH mRNA levels. The ΔΔCq method was used to calculate relative gene expression.

### ChIP assay

The ChIP assay was performed by using the ChIP-IT Express Enzymatic Chromatin Immunoprecipitation kit (Active Motif) according to the manufacturer's instructions. Eluted DNA was amplified by qPCR with GTSE1 promoter-specific primers (sequences shown in Supplementary Table 4).

A mix of two antibodies was used for E2F1 ChIP experiments. Antibodies and dilutions used are reported in Supplementary Table 2.

### Cell migration assays

For the wound-healing assay, silenced cells were plated in 6-well plates and cultured to confluence. Cell monolayers were scratched using a pipette tip, washed with PBS to remove debris, and incubated for 16 hours in cell culture medium. The wounded areas were imaged immediately after wounding and after 16 hours.

For the transwell migration assay, 48 hours after silencing of TEAD1/3/4, 5×10^4^ cells were resuspended in serum-free medium and seeded on the top membrane of 24well 8μm PET cell culture inserts (BD Falcon). Complete medium containing 10% FBS was added to the lower compartment to act as chemoattractant and cells were allowed to migrate for 16 hours at 37°C. After removing unmigrated cells with a cotton swab, migrated cells were fixed in 3% para-formaldehyde (PFA) and stained with crystal violet 0.5%. The migrated cells were then counted in ten randomized fields.

Transwell invasion assays were performed in 24well 8μm PET cell culture inserts (BD Falcon) coated with Corning Matrigel Matrix. 48 hours after silencing, 8×10^4^ cells were plated in cell culture inserts and allowed to invade for 18 hours at 37°C. The invasive cells were fixed in 3% PFA, stained with crystal violet 0.5% and ten randomized fields were counted.

All the migration and invasion assays were performed in triplicate.

### Cell protrusions assay

The cell protrusions assay was performed as previously described [50]. Briefly, after GTSE1 or TEAD1/3/4 silencing by siRNA 1×10^6^ cells were plated in 24well 1μm PET cell culture inserts (BD Falcon) for 4 hours at 37°C. Cells were then fixed in 3% PFA, cell protrusions were stained using F-432 (Molecular Probes) and nuclei were stained using a propidium iodide solution (P4864, Sigma Aldrich). Multiple images of stained nuclei and pseudopodia were taken and counted using ImageJ software. The average number of cell protrusions/cell was obtained dividing the number of pseudopodia by the number of cell nuclei. A three-dimensional representation of cell protrusions was obtained using the Interactive 3D Surface Plot plugin (https://imagej.nih.gov/ij/plugins/surface-plot-3d.html) for ImageJ. Data were obtained from three independent experiments.

### Cell proliferation assay

For cell proliferation assay, 48 hours after TEAD1/3/4 silencing by siRNA, 1×10^5^ cells were plated and counted after 16 hours. Data are shown as mean ± SEM of three independent experiments. For the statistical analysis, Student two-tailed t-test was applied.

### Bioinformatics analysis

Gene expression data and clinical annotation for Breast Cancer samples (TCGA data set) were obtained from the Memorial Sloan Kettering Cancer Genomics Portal (http://www.cbioportal.org/public-portal; last accessed 10 May 2017). Starting from the Breast Invasive Carcinoma dataset (TCGA, Provisional, n=1100) we selected all the patients or the “basal-like” patients with ER, PR and HER2 negative status obtaining a subset of putative TNBC samples (n=107). Based on the RNA-Seq V2 gene expression data, we downloaded the correlation for each gene (n=8263) with respect to GTSE1 expression. Since we observed that there is strong linear correlation between TNBC and ALL BC dataset (cor=0.637, p-value < 2.2e-16), in order to have a stronger support to our analyses we decided to use for the subsequent steps all the breast cancer samples data (n=1100).

The genomic regions surrounding the FANTOM5 CAGE peaks associated with the GTSE1 gene were analyzed using the ExPlain 3.1 suite (http://explain31.biobase-international.com/, MATCH tool) for the presence of transcription factor binding sites (TFBS) possibly involved in GTSE1 gene expression regulation (hg19, chr22 46,692,751-46,692,850, strand=+).

The analysis was performed on the whole region encompassing the FANTOM5 peaks, along with the 1800nt upstream, in order to identify all the transcription factors (TFs) possibly involved in broad regulatory events; this approach allowed us to identify the list of significantly enriched TFs with respect to background regions.

We finally compared this list with the genes co-expressed with GTSE1 in TCGA and the TFs gene list obtained by the FANTOM5 project, ending up with three proteins (TEADs family, HMGA1, and E2F1) corresponding to the most interesting putative regulators of GTSE1 expression.

### Breast cancer dataset subtypes and survival analyses

To verify the correlation of GTSE1 expression and breast cancer clinical data, KM curves for the OS, DMFS, and RFS of breast cancer patients, classified according to the expression were obtained using the Cancer Gene-Expression Miner v4.0 web tool (molecular subtype prognostic analysis module, bc-GenExMiner v4.0) [43,44]. The samples were split into two groups according to the mean expression. To survey the correlation of GTSE1, TEAD4 and E2F1 expression with breast cancer subtype, we utilized the molecular subtype boxplot module. For the univariate cox proportional hazards analyses, we used the Molecular subtype prognostic analysis tool that permits to assess the prognostic impact of a gene within groups of patients with a certain molecular subtype. When possible, we reported the results for all the subtypes as defined by Single Sample Predictors (SSP) and/or Subtype Clustering Models (SCM).

## SUPPLEMENTARY FIGURES AND TABLES



## Abbreviations

ACTB: Actin Beta (official gene symbol ACTB)
BIRC5: Baculoviral IAP Repeat Containing 5 (official gene symbol BIRC5)
CCNE1: Cyclin E1 (official gene symbol CCNE1)
CTGF: Connective Tissue Growth Factor (official gene symbol CTGF)
E2F1: E2F transcription factor 1 (official gene symbol E2F1)
GTSE1: G2 and S phase expressed1 (official gene symbol GTSE1)
HMGA1: High Mobility Group AT-Hook protein 1 (official gene symbol HMGA1)
MCM6: Minichromosome Maintenance Complex Component 6 (official gene symbol MCM6)
MVA: Mevalonic Acid
pRb: Retinoblastoma protein (official gene symbol RB1)
p-pRb: phosphorylated Retinoblastoma protein
HMMR: Hyaluronan-Mediated Motility Receptor (official gene symbol HMMR)
HR: hazard ratio (values are rounded to 2 decimal places)
MSP: Molecular Subtype Predictor (SSPs + SCMs)
RMSPC: Robust Molecular Subtype Predictors Classification based on patients classified in the same subtype with the six MSPs
RSCMC: Robust SCM Classification based on patients classified in the same subtype with the three SCMs
RSSPC: Robust SSP Classification based on patients classified in the same subtype with the three SSPs
SCM: Subtype Clustering Model
SSP: Single Sample Predictor
TAZ: Transcriptional Coactivator With PDZ-Binding Motif (official gene symbol WWTR1)
TEAD: TEA Domain transcription factor (official gene symbol TEAD)
TF: Transcription Factor
TFBS: Transcription Factor Binding Site
TNBC: Triple Negative Breast Cancer
TSS: Transcription Start Site
YAP: Yes-Associated Protein (official gene symbol YAP1)

## References

[1] Weigelt B, Peterse JL, van't Veer LJ (2005). Breast cancer metastasis: markers and models. Nat Rev Cancer.

[2] Caldas C, Stingl J (2007). Molecular heterogeneity of breast carcinomas and the cancer stem cell hypothesis. Nat Rev Cancer.

[3] Mayer IA, Abramson VG, Lehmann BD, Pietenpol JA (2014). New strategies for triple-negative breast cancer-deciphering the heterogeneity. Clin Cancer Res.

[4] Scolz M, Widlund PO, Piazza S, Bublik DR, Reber S, Peche LY, Ciani Y, Hubner N, Isokane M, Monte M, Ellenberg J, Hyman AA, Schneider C (2012). GTSE1 Is a microtubule plus-end tracking protein that regulates eb1-dependent cell migration. PLoS One.

[5] Utrera R, Collavin L, Lazarević D, Delia D, Schneider C (1998). A novel p53-inducible gene coding for a microtubule-localized protein with G2-phase-specific expression. EMBO J.

[6] Collavin L, Monte M, Verardo R, Pfleger C, Schneider C (2000). Cell-cycle regulation of the p53-inducible gene B99. FEBS Lett.

[7] Monte M, Collavin L, Lazarevic D, Utrera R, Dragani TA, Schneider C (2000). Cloning, chromosome mapping and functional characterization of a human homologue of murine Gtse-1 (B99) gene. Gene.

[8] Bublik DR, Scolz M, Triolo G, Monte M, Schneider C (2010). Human GTSE-1 regulates p21CIP1/WAF1 stability conferring resistance to paclitaxel treatment. J Biol Chem.

[9] Subhash VV, Tan SH, Tan WL, Yeo MS, Xie C, Wong FY, Kiat ZY, Lim R, Yong WP, Bilici A, Siegel R, Naishadham D, Jemal A (2015). GTSE1 expression represses apoptotic signaling and confers cisplatin resistance in gastric cancer cells. BMC Cancer.

[10] Bendre S, Rondelet A, Hall C, Schmidt N, Lin YC, Brouhard GJ, Bird AW (2016). GTSE1 tunes microtubule stability for chromosome alignment and segregation by inhibiting the microtubule depolymerase MCAK. J Cell Biol.

[11] Lee J, Sung CO, Lee EJ, Do IG, Kim HC, Yoon SH, Lee WY, Chun HK, Kim KM, Park YS (2012). Metastasis of neuroendocrine tumors are characterized by increased cell proliferation and reduced expression of the atm gene. PLoS One.

[12] Zhou X, Temam S, Oh M, Pungpravat N, Huang BL, Mao L, Wong DT (2006). Global expression-based classification of lymph node metastasis and extracapsular spread of oral tongue squamous cell carcinoma. Neoplasia.

[13] Guo L, Zhang S, Zhang B, Chen W, Li X, Zhang W, Zhou C, Zhang J, Ren N, Ye Q (2016). Silencing GTSE-1 expression inhibits proliferation and invasion of hepatocellular carcinoma cells. Cell Biol Toxicol.

[14] Tian W, Yu J, Tomchick DR, Pan D, Luo X (2010). Structural and functional analysis of the YAP-binding domain of human TEAD2. Proc Natl Acad Sci U S A.

[15] Xiao JH, Davidson I, Matthes H, Garnier JM, Chambon P (1991). Cloning, expression, and transcriptional properties of the human enhancer factor TEF-1. Cell.

[16] Pobbati AV, Hong W (2013). Emerging roles of TEAD transcription factors and its coactivators in cancers. Cancer Biol Ther.

[17] Lamar JM, Stern P, Liu H, Schindler JW, Jiang ZG, Hynes RO (2012). The Hippo pathway target, YAP, promotes metastasis through its TEAD-interaction domain. Proc Natl Acad Sci U S A.

[18] Zhang H, Liu CY, Zha ZY, Zhao B, Yao J, Zhao S, Xiong Y, Lei QY, Guan KL (2009). TEAD transcription factors mediate the function of TAZ in cell growth and epithelial-mesenchymal transition. J Biol Chem.

[19] Wang Z, Wu Y, Wang H, Zhang Y, Mei L, Fang X, Zhang X, Zhang F, Chen H, Liu Y, Jiang Y, Sun S, Zheng Y (2014). Interplay of mevalonate and Hippo pathways regulates RHAMM transcription via YAP to modulate breast cancer cell motility. Proc Natl Acad Sci U S A.

[20] Chen J, Zhu F, Weaks RL, Biswas AK, Guo R, Li Y, Johnson DG (2011). E2F1 promotes the recruitment of DNA repair factors to sites of DNA double-strand breaks. Cell Cycle.

[21] Kel AE, Kel-Margoulis OV, Farnham PJ, Bartley SM, Wingender E, Zhang MQ (2001). Computer-assisted identification of cell cycle-related genes: new targets for E2F transcription factors. J Mol Biol.

[22] Lazzerini Denchi E, Helin K (2005). E2F1 is crucial for E2F-dependent apoptosis. EMBO Rep.

[23] Li FX, Zhu JW, Tessem JS, Beilke J, Varella-Garcia M, Jensen J, Hogan CJ, DeGregori J (2003). The development of diabetes in E2f1/E2f2 mutant mice reveals important roles for bone marrow-derived cells in preventing islet cell loss. Proc Natl Acad Sci U S A.

[24] Luo W, Li G, Yi Z, Nie Q, Zhang X (2016). E2F1-miR-20a-5p/20b-5p auto-regulatory feedback loop involved in myoblast proliferation and differentiation. Sci Rep.

[25] Ishida S, Huang E, Zuzan H, Spang R, Leone G, West M, Nevins JR (2001). Role for E2F in control of both DNA replication and mitotic functions as revealed from DNA microarray analysis. Mol Cell Biol.

[26] Müller H, Bracken AP, Vernell R, Moroni MC, Christians F, Grassilli E, Prosperini E, Vigo E, Oliner JD, Helin K (2001). E2Fs regulate the expression of genes involved in differentiation, development, proliferation, and apoptosis. Genes Dev.

[27] Wells J, Boyd KE, Fry CJ, Bartley SM, Farnham PJ (2000). Target gene specificity of E2F and pocket protein family members in living cells. Mol Cell Biol.

[28] Martelli F, Livingston DM (1999). Regulation of endogenous E2F1 stability by the retinoblastoma family proteins. Proc Natl Acad Sci U S A.

[29] Matys V, Kel-Margoulis OV, Fricke E, Liebich I, Land S, Barre-Dirrie A, Reuter I, Chekmenev D, Krull M, Hornischer K, Voss N, Stegmaier P, Lewicki-Potapov B (2006). TRANSFAC and its module TRANSCompel: transcriptional gene regulation in eukaryotes. Nucleic Acids Res.

[30] Wang C, Nie Z, Zhou Z, Zhang H, Liu R, Wu J, Qin J, Ma Y, Chen L, Li S, Chen W, Li F, Shi P (2015). The interplay between TEAD4 and KLF5 promotes breast cancer partially through inhibiting the transcription of p27Kip1. Oncotarget.

[31] Sorrentino G, Ruggeri N, Specchia V, Cordenonsi M, Mano M, Dupont S, Manfrin A, Ingallina E, Sommaggio R, Piazza S, Rosato A, Piccolo S, Del Sal G (2014). Metabolic control of YAP and TAZ by the mevalonate pathway. Nat Cell Biol.

[32] Bischoff H, Angerbauer R, Bender J, Bischoff E, Faggiotto A, Petzinna D, Pfitzner J, Porter MC, Schmidt D, Thomas G (1997). Cerivastatin: pharmacology of a novel synthetic and highly active HMG-CoA reductase inhibitor. Atherosclerosis.

[33] Siew WC, Chun JL, Guo K, Chee PN, Lee I, Hunziker W, Zeng Q, Hong W (2008). A role for TAZ in migration, invasion, and tumorigenesis of breast cancer cells. Cancer Res.

[34] Etienne-Manneville S (2008). Polarity proteins in migration and invasion. Oncogene.

[35] Sahai E, Marshall CJ (2003). Differing modes of tumour cell invasion have distinct requirements for Rho/ROCK signalling and extracellular proteolysis. Nat Cell Biol.

[36] Zanconato F, Forcato M, Battilana G, Azzolin L, Quaranta E, Bodega B, Rosato A, Bicciato S, Cordenonsi M, Piccolo S (2015). Genome-wide association between YAP/TAZ/TEAD and AP-1 at enhancers drives oncogenic growth. Nat Cell Biol.

[37] Nicolay BN, Bayarmagnai B, Islam AB, Lopez-Bigas N, Frolov MV (2011). Cooperation between dE2F1 and Yki/Sd defines a distinct transcriptional program necessary to bypass cell cycle exit. Genes Dev.

[38] Kapoor A, Yao W, Ying H, Hua S, Liewen A, Wang Q, Zhong Y, Wu CJ, Sadanandam A, Hu B, Chang Q, Chu GC, Al-Khalil R (2014). Yap1 activation enables bypass of oncogenic KRAS addiction in pancreatic cancer. Cell.

[39] Fry DW, Harvey PJ, Keller PR, Elliott WL, Meade M, Trachet E, Albassam M, Zheng X, Leopold WR, Pryer NK, Toogood PL (2004). Specific inhibition of cyclin-dependent kinase 4/6 by PD 0332991 and associated antitumor activity in human tumor xenografts. Mol Cancer Ther.

[40] Finn RS, Dering J, Conklin D, Kalous O, Cohen DJ, Desai AJ, Ginther C, Atefi M, Chen I, Fowst C, Los G, Slamon DJ (2009). PD 0332991, a selective cyclin D kinase 4/6 inhibitor, preferentially inhibits proliferation of luminal estrogen receptor-positive human breast cancer cell lines in vitro. Breast Cancer Res.

[41] Gelbert LM, Cai S, Lin X, Sanchez-Martinez C, Del Prado M, Lallena MJ, Torres R, Ajamie RT, Wishart GN, Flack RS, Neubauer BL, Young J, Chan EM (2014). Preclinical characterization of the CDK4/6 inhibitor LY2835219: In-vivo cell cycle-dependent/independent anti-tumor activities alone/in combination with gemcitabine. Invest New Drugs.

[42] Singh S, Davis R, Alamanda V, Pireddu R, Pernazza D, Sebti S, Lawrence N, Chellappan S (2010). Rb–Raf-1 Interaction Disruptor RRD-251 Induces Apoptosis in Metastatic Melanoma Cells and Synergizes with Dacarbazine. Mol Cancer Ther.

[43] Jézéquel P, Campone M, Gouraud W, Guérin-Charbonnel C, Leux C, Ricolleau G, Campion L (2012). Bc-GenExMiner: An easy-to-use online platform for gene prognostic analyses in breast cancer. Breast Cancer Res Treat.

[44] Jézéquel P, Frénel JS, Campion L, Guérin-Charbonnel C, Gouraud W, Ricolleau G, Campone M (2013). bc-GenExMiner 3.0: New mining module computes breast cancer gene expression correlation analyses. Database.

[45] Bartucci M, Dattilo R, Moriconi C, Pagliuca A, Mottolese M, Federici G, Benedetto AD, Todaro M, Stassi G, Sperati F, Amabile MI, Pilozzi E, Patrizii M (2014). TAZ is required for metastatic activity and chemoresistance of breast cancer stem cells. Oncogene.

[46] Ertel A, Dean JL, Rui H, Liu C, Witkiewicz AK, Knudsen KE, Knudsen ES (2010). RB-pathway disruption in breast cancer: differential association with disease subtypes, disease-specific prognosis and therapeutic response. Cell Cycle.

[47] Qin G, Xu F, Qin T, Zheng Q, Shi D, Xia W, Tian Y, Tang Y, Wang J, Xiao X, Deng W, Wang S (2015). Palbociclib inhibits epithelial-mesenchymal transition and metastasis in breast cancer via c-Jun/COX-2 signaling pathway. Oncotarget.

[48] Brocher J, Vogel B, Hock R (2010). HMGA1 down-regulation is crucial for chromatin composition and a gene expression profile permitting myogenic differentiation. BMC Cell Biol.

[49] Pegoraro S, Ros G, Piazza S, Sommaggio R, Ciani Y, Rosato A, Sgarra R, Del Sal G, Manfioletti G (2013). HMGA1 promotes metastatic processes in basal-like breast cancer regulating EMT and stemness. Oncotarget.

[50] Shankar J, Nabi IR (2011). RNA purification from tumor cell protrusions using porous polycarbonate filters. Methods Mol Biol.

