# Supplementary material for: GTSE1: a novel TEAD4-E2F1 target gene involved in cell protrusions formation in triple-negative breast cancer cell models
Source: Oncotarget. 2017 Jun 27;8(40):67422–38. doi: 10.18632/oncotarget.18691 (PMC5620183; doi:10.18632/oncotarget.18691)
Supplement: Supplementary file 1 [file oncotarget-08-67422-s001.pdf]

GTSE1: a novel TEAD4-E2F1 target gene involved in cell protrusions formation in triple-negative breast cancer cell models

SUPPLEMENTARY MATERIALS

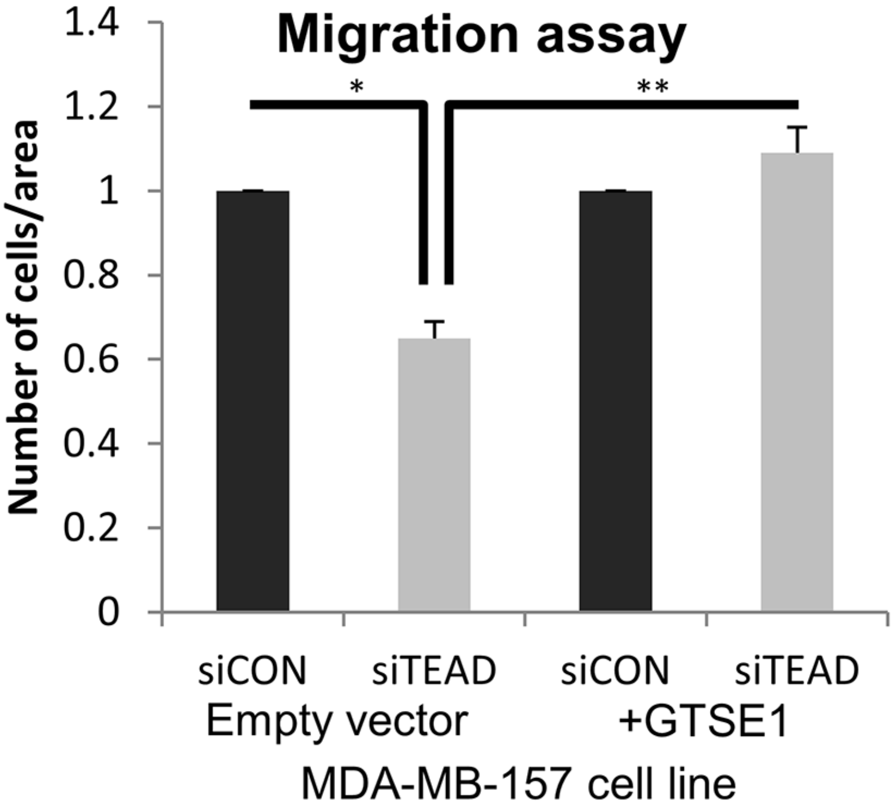

Supplementary Figure 1: The TEAD TF controls breast cancer cell migration in a GTSE1-dependent manner. Analysis of the transwell migration assays showing the ability of GTSE1 to rescue the reduced cell migration after TEAD1/3/4 knockdown. The transwell migration assays were performed in the MDA-MB-157 cell line containing a stably integrated GTSE1 over-expressing construct (pBABE-GTSE1) or an empty vector (pBABE). Histograms show the mean number of cells/area that migrated through the transwell inserts after 16 h. Error bars represent the standard error of the mean from three independent experiments. Student two-tailed t-test was applied for statistical analysis. *p-value<0.05; **p-value<0.01.

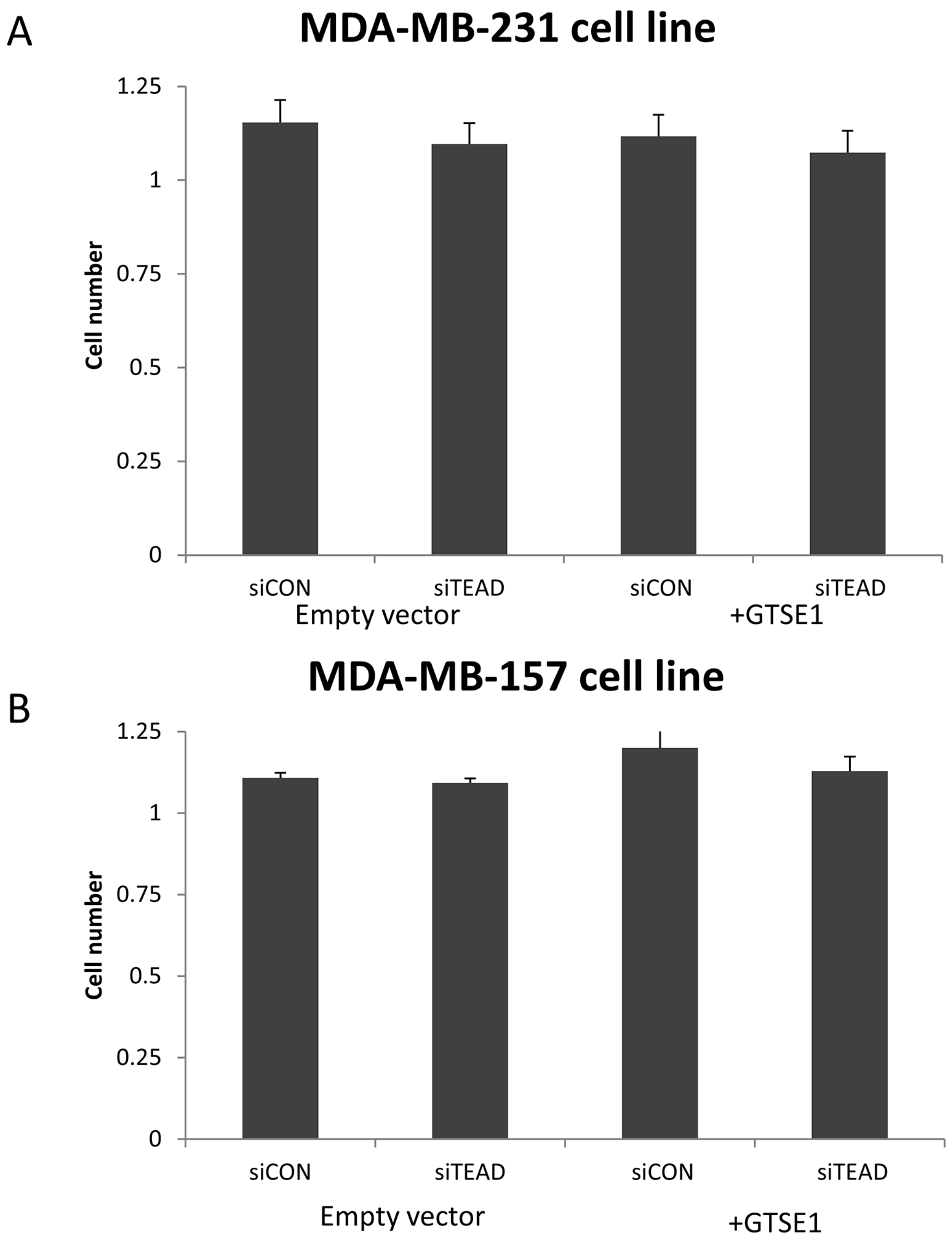

Supplementary Figure 2: GTSE1 rescues the reduced motility observed after TEAD 1/3/4 silencing without any significant effect on the total number of cells in the considered time interval. The cell proliferation assays were carried out in the MDA-MB-231 **(A)** and MDA-MB-157 **(B)** cell lines containing a stably integrated GTSE1 over-expressing construct (pBABE-GTSE1) or an empty vector (pBABE). 48 hours after TEAD1/3/4 silencing, $1X10^5$ cells were plated and counted after 16 hours. Data are shown as mean ± SEM of three independent experiments. For statistical analysis Student two-tailed t-test was applied.

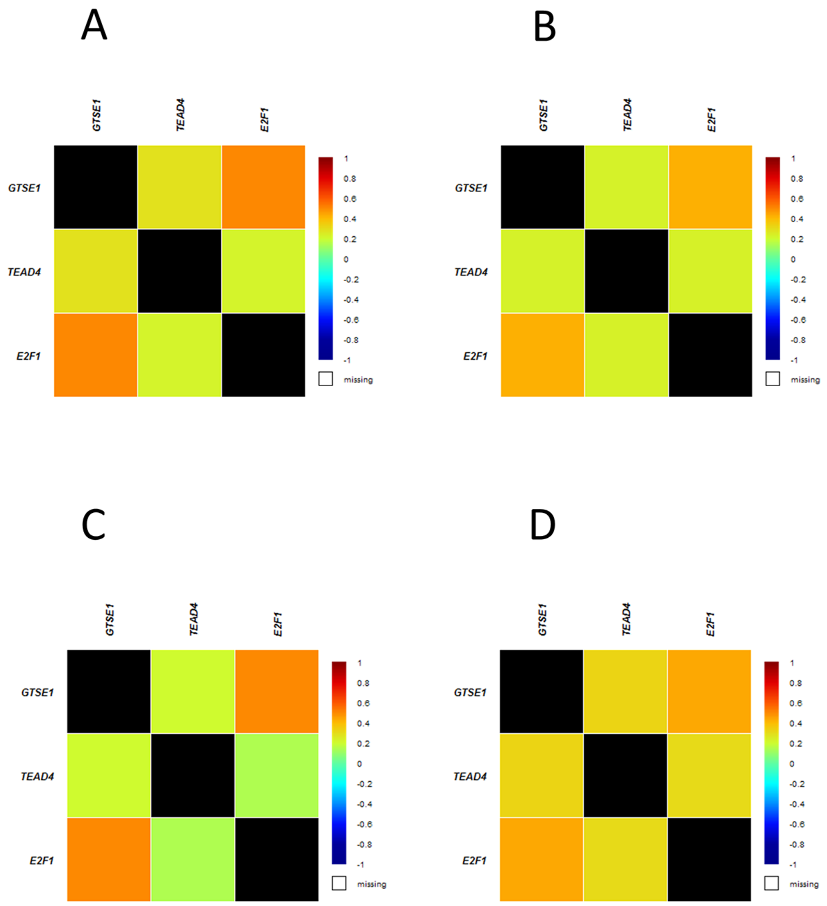

Supplementary Figure 3: Correlation heatmaps of the expression levels of GTSE1, E2F1 and TEAD4. Correlation heatmaps of the expression levels of GTSE1, E2F1, TEAD4 in the different breast cancer subtypes: **(A)** all samples, **(B)** Basal, **(C)** ER negative, **(D)** ER positive.

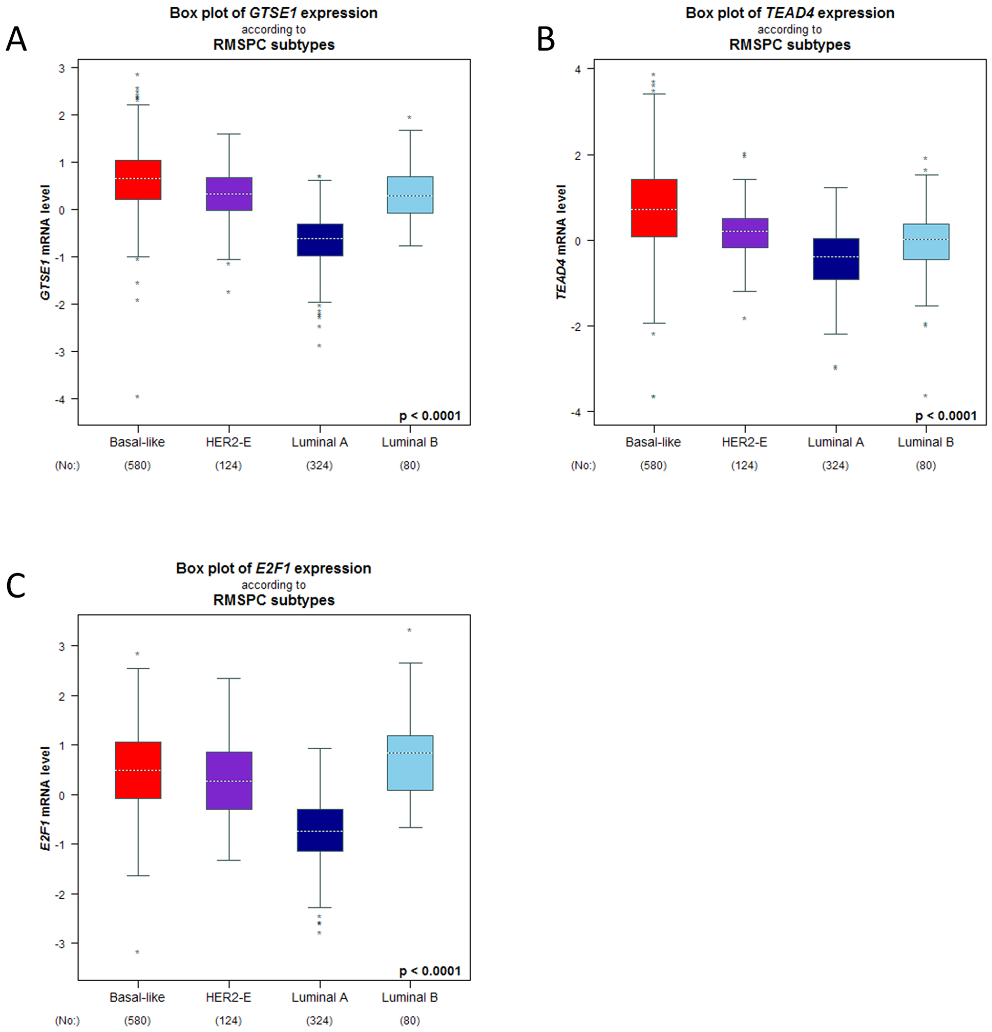

Supplementary Figure 4: Boxplots of the expression levels of GTSE1, E2F1 and TEAD4. Boxplot representations of the expression levels of **(A)** GTSE1, **(B)** E2F1 and **(C)** TEAD4 in the different breast cancer subtypes (robust molecular subtype predictor classification (RMSPC)).

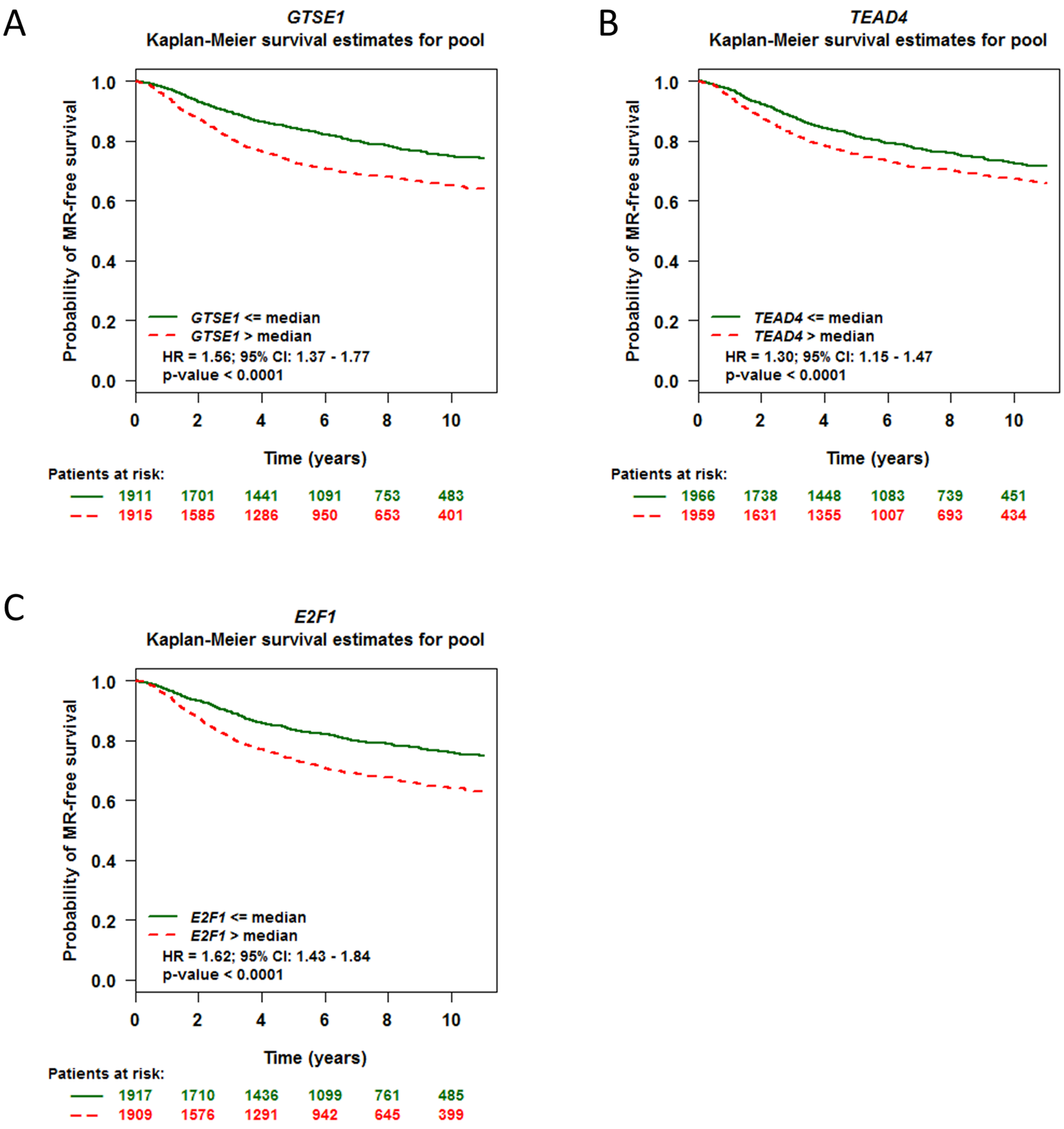

Supplementary Figure 5: Survival analyses based on GTSE1, E2F1 and TEAD4 expression levels. Kaplan–Meier survival curves according to GTSE1, E2F1 and TEAD4 expression levels. Kaplan–Meier survival analyses were performed using the Targeted prognostic analysis module of Breast Cancer Gene-Expression Miner v4.0 (bc-GenExMiner v4.0) in all breast cancer populations and using Metastatic relapse (MR) as event type. Red/Blue lines represent higher/lower level of expression of **(A)** GTSE1, **(B)** TEAD4 and **(C)** E2F1.

SCM molecular subtype	Molecular subtype		ER-/HER2-			HER2-E			ER+/HER2- low proliferation			ER+/HER2- high proliferation		
	Analysis results		p-value	HR	95% CI	p-value	HR	95% CI	p-value	HR	95% CI	p-value	HR	95% CI
GTSE1	Single Sample Predictors	Sorlie's	0.6949	0.97	0.85 - 1.12	0.0020	1.38	1.12 - 1.68	0.0085	1.27	1.06 - 1.52	0.0005	1.29	1.12 - 1.50
		Hu's	0.6660	0.97	0.83 - 1.13	0.0004	1.40	1.16 - 1.70	0.0241	1.19	1.02 - 1.39	0.0001	1.35	1.16 - 1.56
		PAM50	0.0030	1.15	1.05 - 1.26	0.2240	1.12	0.93 - 1.35	0.0003	1.56	1.22 - 1.98	0.0091	1.27	1.06 - 1.53
	RSSPC		0.2341	0.90	0.75 - 1.07	0.0438	1.34	1.01 - 1.78	0.0739	1.36	0.97 - 1.91	0.4941	1.08	0.86 - 1.37
	RMSPC		0.2848	0.89	0.71 - 1.11	0.8291	0.93	0.51 - 1.73	0.5704	1.17	0.67 - 2.04	0.6851	0.87	0.45 - 1.70
TEAD4	Single Sample Predictors	Sorlie's	0.2858	1.06	0.95 - 1.17	0.0446	1.19	1.00 - 1.40	0.0012	1.25	1.09 - 1.43	0.2813	1.05	0.96 - 1.16
		Hu's	0.0553	1.11	1.00 - 1.23	0.0589	1.14	0.99 - 1.31	0.1605	1.10	0.96 - 1.26	0.1284	1.08	0.98 - 1.19
		PAM50	0.0085	1.11	1.03 - 1.20	0.0587	1.13	1.00 - 1.28	0.3257	1.09	0.91 - 1.31	0.0850	1.11	0.99 - 1.26
	RSSPC		0.2819	1.07	0.95 - 1.21	0.0238	1.35	1.04 - 1.75	0.0957	1.27	0.96 - 1.67	0.4238	1.06	0.92 - 1.23
	RMSPC		0.7401	1.02	0.89 - 1.18	0.2363	1.35	0.82 - 2.23	0.5494	1.12	0.77 - 1.65	0.6693	1.12	0.67 - 1.85
E2F1	Single Sample Predictors	Sorlie's	0.7763	0.98	0.86 - 1.12	0.0071	1.25	1.06 - 1.47	0.1053	1.13	0.98 - 1.30	0.0411	1.11	1.00 - 1.23
		Hu's	0.6697	1.03	0.90 - 1.18	0.0009	1.28	1.11 - 1.48	0.0913	1.13	0.98 - 1.30	0.1223	1.08	0.98 - 1.20
		PAM50	0.0017	1.14	1.05 - 1.25	0.7339	0.98	0.85 - 1.12	0.0124	1.27	1.05 - 1.54	0.0010	1.24	1.09 - 1.41
	RSSPC		0.4768	0.94	0.80 - 1.11	0.1890	1.18	0.92 - 1.51	0.2125	1.20	0.90 - 1.59	0.0446	1.18	1.00 - 1.39
	RMSPC		0.4621	0.93	0.76 - 1.13	0.1886	1.36	0.86 - 2.16	0.2394	1.27	0.85 - 1.89	0.5079	1.16	0.75 - 1.77

Supplementary Figure 6: Cox models of GTSE1, E2F and TEAD4. Univariate cox proportional hazards analyses for GTSE1, E2F and TEAD4 in the different breast cancer SCM (Subtype Clustering Models) subtypes. Several molecular classifications are presented: Single Sample Predictor (SPP), Robust SSP Classification Robust Molecular (RSSPC) and Robust Molecular Subtype Predictors Classification (RMSPC). P-values were colored accordingly to their statistical significance (green $p < 0.05$; orange $0.05 < p < 0.1$; red $p > 0.1$).

Supplementary Table 1: siRNAs sequences. Table listing the sequences of the siRNAs used for the RNA interference experiments.

siRNA	Sequence
GTSE1	5’-GAUUCAUACAGGAGUCAAATT-3’
TEAD1/3/4	5’-UGAUCAACUUCAUCCACAAGC-3’
YAP	5’-GACAUCUUCUGGUCAGAGA-3’
TAZ	5’-ACGUUGACUUAGGAACUUU-3’
E2F1	5’-CCAACGUCCUUGAGGGCAU-3’,
	5’-CUGCAGAGCAGAUGGUUAU-3’
	5’-GGAAAGUGAGGGAGGGAGA-3’

Supplementary Table 2: Antibodies and dilutions. Table listing the antibodies and the dilutions used in the Western blot and ChIP experiments.

Target	Western blot Antibodies	Dilution
ACTB	C11, Sigma Aldrich	1:3000
BIRC5	sc-10811, Santa Cruz Biotechnology	1:1000
E2F1	sc-251, Santa Cruz Biotechnology	1:1000
GTSE1	Rabbit Home made	1:500
HMMR	ab108339,Abcam	1:1000
Pan-TEAD	13295, Cell Signaling	1:2000
pRb	554136, BD Pharmingen	1:1000
p-pRb	ab76298, Abcam	1:1000
YAP/TAZ	sc-101199, Santa Cruz Biotechnology	1:1000
**Target**	**ChIP Antibodies**	**Concentration**
E2F1	sc-251, Santa Cruz Biotechnology	5ng/µl
	sc-193, Santa Cruz Biotechnology	5ng/µl
TEAD4	5H3, Abnova	10ng/µl

Supplementary Table 3: RT-qPCR primer sequences. Table reporting the sequences of the primers used for RT-qPCR experiments.

Target gene	Primer sequence
GTSE1	FW 5’-GCCCCGGGTGCTGTCAATGT-3’
	Rev 5’-GCCCACTGCTGGGGATGTGC-3’
TEAD4	FW 5’-TTGGAACTGGCTTAGCGCAC-3’
	Rev 5’-CGTCATTGTCGATGGGCTTG-3’
YAP1	FW 5’-GCCGGAGCCCAAATCC-3’
	Rev 5’-GCAGAGAAGCTGGAGAGGAATG-3’
TAZ	FW 5’-CAGCAATGTGGATGAGATGG-3’
	Rev 5’-TCATTGAAGAGGGGGATCAG-3’
E2F1	FW 5’-AACATCGATCGGGCCTTGTTTG-3’
	Rev 5’-GTGGACTCTTCGGAGAACTTTCAG-3’
BIRC5	FW 5’-AGCATTCGTCCGGTTGCGCT-3’
	Rev 5’-TCGATGGCACGGCGCACTTT-3’
RHAMM	FW 5’-AGAACCAACTCAAGCAACAGG-3’
	Rev 5’-AGGAGACGCCACTTGTTAATTTC-3’
CTGF	FW 5’-AGGAGTGGGTGTGTGACGA-3’
	Rev 5’-CCAGGCAGTTGGCTCTAATC-3’
CCNE1	FW 5’-TGAGCCGAGCGGTAGCTGGT-3’
	Rev 5’-GGGCTGGGGCTGCTGCTTAG-3’
MCM6	FW 5’-ATCCCTCTTGCCAAGGATTT-3’
	Rev 5’-GAAAAGTTCCGCTCACAAGC-3’
ACTB	FW5’-CCAACCGCGAGAAGATGA-3’
	Rev 5’-CCAGAGGCGTACAGGGATAG-3’
GAPDH	FW 5’-TCTCTGCTCCTCCTGTTC-3’
	Rev 5’-GCCCAATACGACCAAATCC-3’

Supplementary Table 4: ChIP primer sequences. Table listing the sequences of the primers used for ChIP experiments.

Target	Primer sequence
TEAD4 ChIP site 1	S1-FW 5'-CCACACCTACTATGTGCTGACATG-3'
	S1-Rev 5'-CCTCAGCTCATCCTGGGGATGT-3'
TEAD4 ChIP site 2	S2-FW 5'-GATCCCTCTGCCATTCTCCCATGA-3'
	S2-Rev 5'AGGTGGGTGTGGTCAAACAGCT-3'
TEAD4 ChIP site 1 and 2	S1-FW 5'-CCACACCTACTATGTGCTGACATG-3'
	S1S2-Rev 5'TGGAAAGAGTTTGGCCTGCTCA-3'
E2F1 ChIP site 1	ES1-FW 5'CTACACACAAGGAGCTGCTAT-3'
	ES1-Rev 5'-ATCACCCACCCGGAAGT-3'
E2F1 ChIP site 2	ES2-FW 5'-ATGAGTCTCCCTCAGGTCTC-3'
	ES2-Rev 5'-TAAGGGTGTCGATGGGAAGA-3'
E2F1 ChIP site 3,4 and 5	ES3,4,5-FW 5'-GCTCTCTCCTCCAACGCA-3'
	ES3,4,5-Rev 5'AGAGACCTGAGGGAGACTCA T-3'
